# Small CBPR Grants Program: An Innovative Model to Build Sustainable Academic-Community Partnerships

**DOI:** 10.18060/27204

**Published:** 2023-11-01

**Authors:** Payam Sheikhattari, Jummai Apata, Gillian Beth Silver, Shiva Mehravaran, Emma Mitchell, Shervin Assari

**Affiliations:** 1 Prevention Sciences Research Center, School of Community Health and Policy, Morgan State University; 2 Center for Urban Health Disparities Research and Innovation, Morgan CARES Community Engagement Core, Morgan State University; 3 Department of Biology, Morgan State University; 4 Department of Family Medicine, Charles R Drew University of Medicine and Science; 5 Department of Family Medicine, Charles R Drew University of Medicine and Science

**Keywords:** community-based participatory research (CBPR), capacity development, seed funding, research partnership, partnership readiness

## Abstract

Community-based participatory research (CBPR) is an effective approach for addressing health disparities by integrating diverse knowledge and expertise from both academic and community partners throughout the research process. However, more is needed to invest in the foundational infrastructure and resources that are necessary for building and maintaining lasting trusting research partnerships and supporting them to generate impactful CBPR-based research knowledge and solutions. Small CBPR Grants Program is a CBPR-seed-funding program that may be particularly helpful to minority-serving institutions’ and universities’ goal to invest in genuine community-engaged participatory research. Between 2016 and 2019, the Morgan State University Prevention Sciences Research Center, in collaboration with other community and academic organizations, provided 14 small CBPR awards to new partnerships, and evaluated the success and challenges of the program over a period of three years. To achieve our goal, technical support and training were provided to these partnerships to help with their growth and success. The expected outcomes included trusting relationships and equitable partnerships, as well as publications, presentations, and new proposals and awards to work on mutually identified issues. The program’s resulted in continued partnerships beyond the program (in most cases), a founded CBPR Center namely ASCEND, and several secured additional fundings. Keys to the program’s success were supporting the formation of research partnerships through networking opportunities and information sessions, as well as providing small grants to incentivize the development of innovative concepts and projects. A learning network and local support group were also created to enhance productivity and the overall impact of each project.

## Introduction

Community-Based Participatory Research (CBPR) is an effective approach for addressing health disparities by bridging the gap between research and action (N. B. Wallerstein & Duran, 2006). In CBPR, knowledge is collaboratively produced and owned by a diverse group of stakeholders, including local communities (Israel et al., 1998). Throughout the research process, non-academic stakeholders are empowered and engaged, promoting shared decision-making and co-learning among all involved (Paradiso de Sayu & Chanmugam, 2016; Ross et al., 2010). By involving community members and other stakeholders in the research process, CBPR helps to ensure that research findings are relevant, applicable, and meaningful to the communities, and that the research leads to positive changes that benefit local communities (Skizim et al., 2017; N. Wallerstein & Duran, 2010).

Research partnerships in CBPR are not about transforming community partners into academic researchers, or vice versa. Instead, successful CBPR projects require negotiation and compromise through dialogue and trust-based relationships. Building a strong and trusting partnership between academic researchers and members of the community is crucial, with clarity around each party’s role and how they could benefit from the relationship (Andrews et al., 2013). Underserved communities have unique knowledge and connections to offer, including first-hand experience with health and social issues affecting their communities and the history of actions and solutions adopted to address them. While university researchers may have methodological expertise, analytical skills, and access to research funding, their limited understanding of insider perspectives on community problems can be a challenge. In some cases, researchers lack the first-hand/lived experience needed to truly understand challenges in the communities they are expected to serve. Therefore, creating a collaborative partnership between academic researchers and community members can bring valuable insights that can lead to more relevant and impactful research (Martinez et al., 2013).

Despite the increasing demand, the potential of CBPR to address health disparities remains unrealized, as the traditional university-controlled approach to research remains the norm (Coombe et al., 2023). CBPR projects take longer than traditional studies. The effectiveness and impact of CBPR projects is greater when academic and community partners spend ample time learning from one another, developing agreeable plans, and nurturing productive and trusting partnerships (Jagosh et al., 2015; Ross et al., 2010; N. Wallerstein & Duran, 2010). Building a strong research partnership is challenging, and various roadblocks can hinder progress. Sharing power, nurturing a co-learning environment, and developing capacity are among the most significant challenges (Andress et al., 2020; Coombe et al., 2020; Henry Akintobi et al., 2021; Israel et al., 1998; Muhammad et al., 2015). Developing trusting and inclusive CBPR partnerships, particularly among people who have not worked together previously, requires time and co-learning (e.g., reciprocal exchange of knowledge and skills) (Coombe, C. M., 2023). A range of issues, including low self-confidence, fear, hesitation to participate, and mistrust in research (often rooted in historical events and traumatic experiences), are common challenges related to fostering strong research partnerships between community and academic researchers (Coombe et al., 2023). On the other hand, university researchers’ lack of understanding of the insider perspective on problems may result in research questions that do not align with community priorities, suboptimal utilization of local existing resources and assets, and low levels of participation among populations experiencing health disparities (Sheikhattari et al., 2012). Facilitating communication and teamwork among a diverse group of partners presents logistical challenges related to securing necessary support and resources, scheduling and coordinating activities, record-keeping, documentation, and accountability. Partners who are interested in collaborating on a CBPR project often need support in negotiating their roles, a crucial step needed to begin a successful research partnership. Further, potentially effective researchers may not be very effective because they may not get engaged in research partnerships or may face many challenges related to how to get started. This is in part because researchers may need to gradually build their mastery of the roles required in partnered research (Sheikhattari & Kamangar, 2010).

One solution to promote community-academic partnerships to address health disparities is the provision of “seed funding”. These CBPR small grants boost partnerships, develop methods of engaging the community, and identify shared research priorities (Coombe et al., 2023). These steps are helpful to flush out during a small project, before seeking larger grants with more stringent expectations and timelines. Fostering the relationship takes time, and traditional research partnerships do not account for the additional skills and time that successful CBPR projects require. Factors contributing to the success of CBPR seed funding include the development of operational, training, and mentoring capacity to address challenges. A strong infrastructure that facilitates connections, communication, and innovation enables the development of a network of diverse stakeholders. Co-learning activities, relevant skill-building opportunities, and technical assistance are also essential for creating and sustaining a vibrant ecosystem of CBPR projects and partnerships. However, there are also significant problems associated with seed funding. Institutions and funders rarely provide sufficient time and resources for the critical stage of establishing equitable partnerships, especially beyond the initial funding period. Partner preparedness can significantly impact partnership development and sustainability. Additionally, there is limited evidence on approaches that intentionally combine initial funds, capacity building, and experienced guidance from community-academic partners to improve the effectiveness and sustainability of CBPR partnerships (Coombe et al., 2023)(Thompson et al., 2010)(Jenkins et al., 2020). Overall, seed funding may help establish and support community-academic partnerships, but there is a need for ongoing support and resources to ensure the success and sustainability of such programs (Kegler et al., 2016).

In this paper, we introduce a Small CBPR Grants Program that aimed to create, sustain, and grow academic-community partnerships addressing health disparities of underserved populations. First we present the program’s methods and results. Then we share our lessons learned (e.g., how such an approach requires capacity building and training services). We close this paper with proposing a new evaluation framework and our final thoughts. In our case, this approach led to the establishment of a CBPR Center (i.e., ASCEND), a foundational infrastructure for successful CBPR initiatives and sustainable partnerships.

## Methods

### The ASCEND Small Grants Program

The ASCEND Center, a multi-disciplinary program, was created under the Morgan State University (MSU)’s Division for Research and Economic Development. This housed the ASCEND Small CBPR Grants Program, funded by the National Institutes of Health BUILD initiative, aimed to create capacity for designing and implementing community-oriented research projects at MSU in Baltimore, Maryland (Kamangar et al., 2017). The program provided up to $20,000 in seed funding per project, along with capacity development, training, and technical assistance services. A joint effort between an MSU faculty member and a community investigator was required to apply for the grants. The Morgan State University Prevention Sciences Research Center administered the program, building on over 15 years of successful research partnerships with underserved communities in Baltimore. One such partnership was CEASE, which was created in 2007 to find solutions to tobacco health disparities in urban settings. The CEASE program grew into a multifaceted partnership, including peer-motivation smoking cessation interventions and preventive and policy advocacy initiatives (Petteway et al., 2019; Sheikhattari et al., 2016; Wagner et al., 2016). The small grants program was modeled after past successful initiatives of the CEASE partnership and the Prevention Sciences Research Center (PSRC). It was developed and implemented from 2017 to 2019 to increase the capacity of MSU faculty and students to conduct health research and engage communities in research. The main purpose was to incentivize the development of community-academic partnerships and nurture the formation of an organic local network of CBPR investigators supporting each other and maximizing their overall impact.

### Morgan CARES Network

CARES model, evolved organically in tandem with the program implementation and based on readiness stages, guided the evaluation and capacity building efforts. This was the result of collaboration of our partners, including project group members and the Community University Advisory Board (CUAB), as a larger learning community, to conduct high-quality research that addresses community health and reduces health disparities. The grants awarded for the CBPR projects served as a catalyst for subsequent awards and formed the foundation for the community-engagement core of a new Center for Urban Health Disparities Research and Innovation (Sheikhattari, 2022; Akintobi, 2021). Since 2019, the Morgan CARES Network has been the home to all CBPR partners, many of whom have contributed to shaping the organization’s governing structure and have held key leadership positions. The Morgan CARES Network has also instituted a new seed community awards program, with established partnerships assuming mentoring roles. Several partners have successfully brought in large awards through this program (Sheikhattari et al., 2022).

### Program Design, Oversight, and Financial Management

Similar to many CBPR projects, this was an iterative initiative that developed over time. The program utilized a mixed methods case study design, as outlined by Creswell and Clark (2017) (Creswell & Clark, 2017). This program ran from Spring 2016 to Summer 2019 at MSU. To ensure that the program was culturally sensitive and responsive to the needs of the community, several individuals from diverse community and university backgrounds were recruited to form the Community University Advisory Board (CUAB). The CUAB played a vital role in shaping the program by co-creating and approving the request for proposals (RFP), promoting the program to potential community and academic investigators, identifying review committees, and recommending proposals for NIH funding. Community engagement was also critical in ensuring that the program was successful. Additionally, to promote transparency and ensure shared access and control of program funds, program management was sub-awarded to a community-oriented fiscal agency called Fusion Partnerships, Inc.

### Description of the Grant Application Process

The program utilized a potentially competitive process with three rounds of request for proposals, resulting in the selection of 14 grantees. The implementation of the program involved a comprehensive approach that included a call for proposals, information sessions, proposal review and funding, grantee workshops and technical assistance, and monitoring and evaluation. Successful grantees were further supported in attending national conferences, publishing their results, and applying for other grants to continue their work. The program supported projects that aimed to build equitable partner relationships and explore collaborative research interests in various health-related areas (given the source of funding was NIH). These projects could include, for example, community assessments, health education and promotion, pilot testing of innovative interventions, or evaluation of existing programs and initiatives. The CBPR projects were jointly led by investigators from the university and the community in an intentional design to promote equal power between partners. Each round of the grant program began with the announcement of the funding opportunity on the ASCEND website, MSU campus-wide emails, and through broad dissemination to community networks. Interested applicants contacted the ASCEND program for more information. As an initial step, letters of intent were required for all applicants intending to submit a proposal. The length of the proposal was up to six pages following the NIH format, and the budget was up to $20,000 in direct costs, with travel money for conference participation and presentations provided outside the program upon successful completion of the projects. The program provided matchmaking services, connecting interested community members with appropriate MSU faculty members and vice versa, to support partnerships in developing full proposals. Technical assistance workshops were also provided; 26 partnerships were represented by 33 individuals at these workshops.

### Selection Criteria and Review Process for Proposals in the CBPR Project

Grantees were selected through a rigorous and competitive review process. Each proposal was evaluated by two to three external reviewers, at least one an academician, and one a community member. The comments were then discussed at a CUAB meeting to decide which projects should be recommended to the NIH for funding. In this program, the selection criteria and review process for proposals were crucial components in ensuring the quality and feasibility of the proposed projects. The review process was highly competitive and helped to ensure that the projects were viewed from multiple perspectives and evaluated based on their potential for creating impactful CBPR partnership. The selection criteria for the proposals were focused on building equitable partner relationships, exploring collaborative research interests, and addressing health-related issues in the community. The proposals that best met these criteria were recommended for funding.

### Evaluation and Monitoring of the CBPR Small Grants Program

**FIGURE 2.**
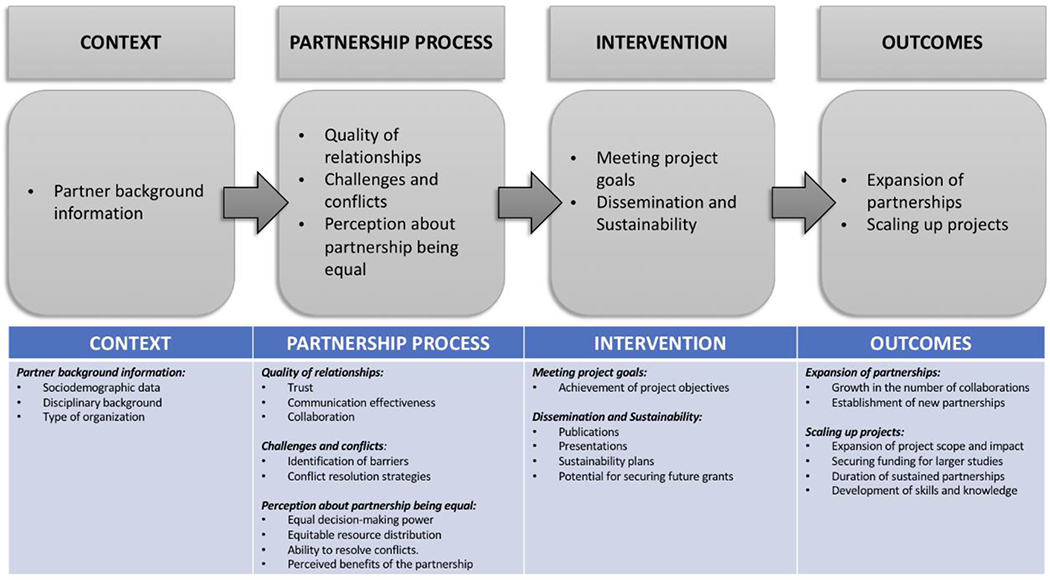
Evaluation model for the CBPR small grant program.

Mixed methods case study design was used for evaluation of the program. The CBPR Small Grants Program was monitored and evaluated using both qualitative and quantitative data collection methods. The monitoring and evaluation team consisted of the program evaluator, principal investigator, program manager and research associate in collaboration with an external team from the University of New Mexico Center for Participatory Research. To enhance the small grants initiative, we consulted the University of New Mexico Center for Participatory Research in 2018, adapted their Engage for Equity (E2) tools (N. Wallerstein et al., 2020), and incorporated the tools into the program monitoring and evaluation. The E2 CBPR model was used for visioning with grantees, and the constructs and metrics of partnering from each of the model’s domains were chosen for evaluating the grantee dynamic partnership processes (Figure 2). The language of items was modified as needed for respondent comprehension, resulting in tailored tools and instruments that would be useful, consistent, and valid for use at different phases of the program. Data were collected through project progress reports submitted by project teams, discussions at workshops, visioning exercises using one of the E2 tools (the River of Life) (N. Wallerstein et al., 2020), individual interviews, and other qualitative assessments.

The collected data included constructs measuring context, partnership processes (partnership experiences, perceptions, power dynamics, and participation), intervention, and outcomes. For the context domain, we captured background information on partnerships such as sociodemographic information, field or discipline of work and the type of organizations from letters of intent and individual interviews. The visioning exercise using the River of Life as well as discussions at workshops were utilized in the partnership process domain to assess the quality of partner relationships using indicators like trust, communication effectiveness and collaboration. In this domain, we also captured challenges and conflicts by identifying barriers and conflict resolution strategies. Additionally, perceptions of the equality in the partnerships were examined by assessing the equality in decision-making power, resource distribution, ability to resolve conflicts and perceived benefits of members of the partnerships. The measures for the intervention domain examined the achievement of project goals and objectives, dissemination and sustainability efforts such as publications and presentations, sustainability plans and the partnership potential for securing future grants. Project progress reports, workshop discussions and in-depth interviews were used to capture these indices of the intervention. For outcomes, using project progress reports and individual interviews, we assessed the expansion of partnerships measured by the growth in number of collaborations and establishment of new partnerships. We also measured the scaling up of projects by assessing the expansion of project scope and impact, securing funding for larger studies, duration of sustained partnerships, development of skills and knowledge.

After data collection, quantitative data were summarized into descriptive tables and figures, while the qualitative data were reviewed and coded based on the themes that emerged. The evaluation process helped to identify areas where the program was successful, as well as areas where improvement was needed. Overall, the evaluation process provided valuable insights that helped to shape and refine the program, making it more effective and responsive to the needs of the community and academic partners. The use of tailored evaluation tools and metrics ensured that the program was able to capture the unique perspectives and experiences of all stakeholders, leading to a more comprehensive and nuanced understanding of the program’s impact.

## Results

### Overview of the Applications and Funded Projects

**FIGURE 3.**
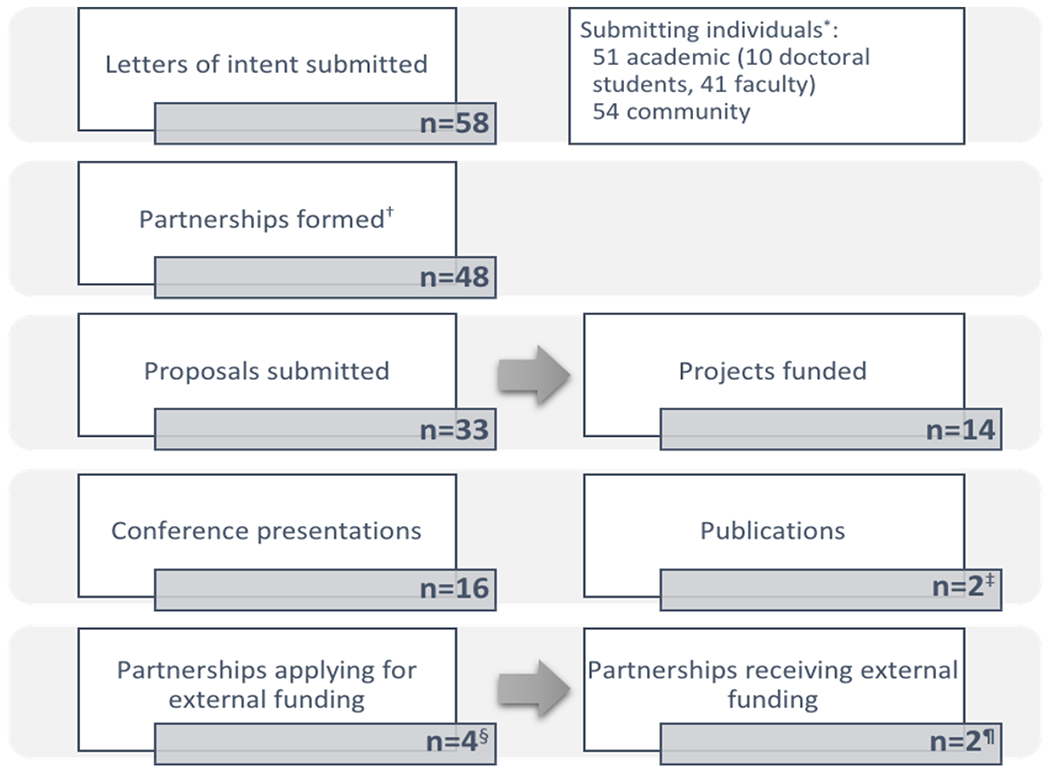
Flow chart showing the process of the CBPR small grants program.

Figure 3 summarizes the program’s outcomes. Our program consisted of three rounds, with a total of 105 individuals (51 academic and 54 community) forming 48 partnerships and submitting 58 letters of intent. Out of these, 33 full proposals were submitted and reviewed. Fourteen projects received up to $20,000 each in seed funding. The remaining 19 un-funded projects received reviewers’ summary statements and guidance for resubmission. The program involved 27 faculty mentors who provided guidance to 20 graduate and 31 undergraduate students from MSU. Funded projects addressed various health-related research topics, including nutrition, tobacco cessation, medical technology, needs assessments, sanitation, built environment, grief support, and mental health, among others.

Table 1 summarizes the characteristics of the principal investigators (PIs) of the 14 funded projects. A total of 29 individuals served as co-principal investigators across projects. Overall, there were more female (n=21; 72.4%) compared to male (n=8; 27.6%) PIs. Most PIs were Black/African American (n=20; 69.0%). A majority of the PIs (65.5%) had no previous experience with CBPR, however, more academic PIs (n=6; 42.9%) reported having some previous exposure to CBPR compared to community PIs (n=4; 26.7%). Previous experience with grant writing was also more common with academic PIs (n=10; 71.4%) compared to community PIs (n=8; 53.3%). Of the 29 PIs, most (62.1%) had some experience applying for grants in the past. As of the time of this report, 16 presentations were made at scientific conferences, two manuscripts were being developed, four of the 14 partnerships had applied for external funding to continue their work in the community, and two of them had been funded.

Table 2 presents a summary of the collaborative activities of the 14 funded project partnerships, documenting their achievements and challenges. Most PIs (71%) believed they have a shared understanding of the program’s goals and mission. Similarly, most partnerships reported equal involvement in the project. Similar results were reported for decision-making power.

## Qualitative Evidence

### Stages of Partnership Readiness

As shown by Table 3, a few stages of partnership readiness were identified through qualitative assessments. Partners with similar levels of readiness reported comparable assets, perceived needs, and recommended services. This table summarizes these stages based on reported qualitative data on the level of partnership experience, knowledge, and readiness in CBPR. The first stage was orientation and connection, which is relevant for junior academic and community investigators with no prior experience in CBPR. This stage involved relationship-building support and opportunities and provided an orientation to the basic foundations of engaging in research partnerships, including roles and contributions. Partners with prior relationships and some experience but without clear ideas and negotiated research concepts were labeled being in the ideation and innovation stage. These were individuals who had formed relationships but needed support to generate novel ideas and write their innovative concepts into a proposal. Partners at the collaboration stage were those with funded projects. Some of the more successful partnerships that completed their projects then progressed to the stages of actively disseminating the results, sustaining their relationships, and planning their next collaborations.

### Capacity Development and Training

Box 1 shows technical workshops to empower CBPR collaboration at MSU with partnering communities. One of the most common services was opportunities for professional networking, match-making recommendations, and general orientation on building trusting research partnerships with those who have complementary knowledge and skills but come from different backgrounds. At this stage, new and established partners were able to connect and start building relationships. According to one academic partner, “Connecting with the community helped them to better understand our [academic] world and vice versa.” Another partner said, “It started off slow, but we eventually got the rhythm.…” Partnerships under development were provided with information sessions and relevant activities to orient and prepare them for participation in the initiative.

**FIGURE 4.**
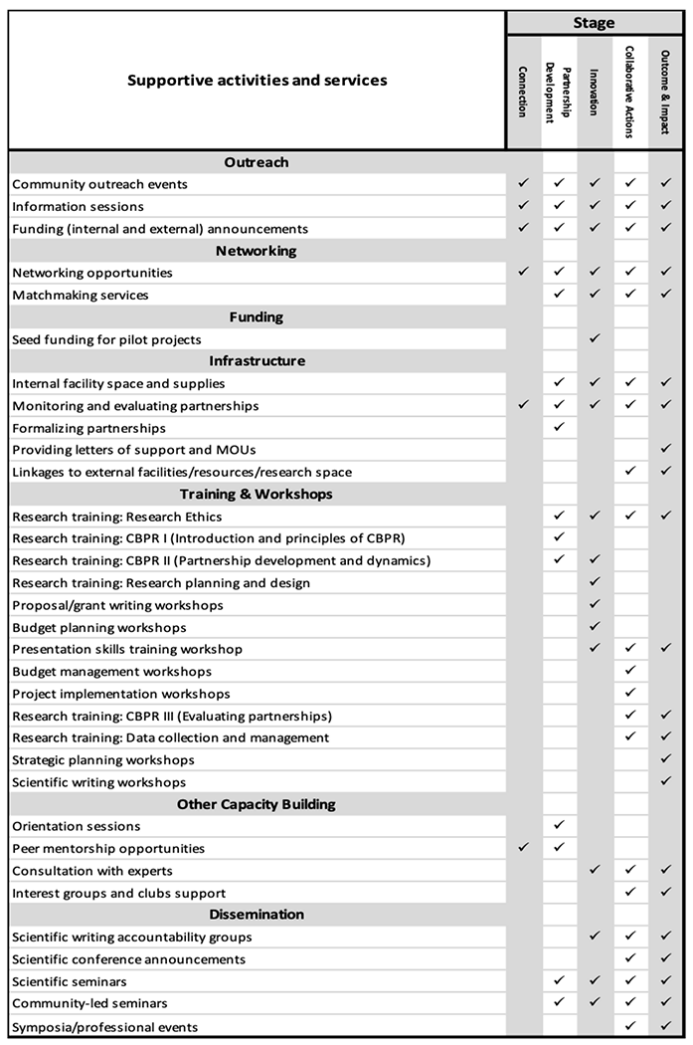
Capacity development and training services by partners stages of readiness.

As shown in Figure 4, newly formed or existing partnerships often requested technical assistance and workshops to develop their proposals and actively collaborate on projects. These services included proposal writing, research budgets, and Institutional Review Board applications, and were offered on a case-by-case basis in informal settings. As one partner expressed, “The support given was great, especially hearing about other people’s experiences in a small group setting.” Grant writing was one of the identified needs, and relevant support was provided to teams. As one partner shared, “This initiative helped me face my fears regarding grant writing and grant management. It lifted my confidence in my ability to implement a research project and taught me valuable skills in doing so.”

Regarding equal partnership, one partner commented, “We worked well together, and everyone’s roles were complimentary; we understood the objectives, and we were on the same page.” As the changing needs at this level require more specialized assistance, plenary discussions, and reflection, renowned CBPR expert consultants were brought in to enhance equitable collaboration. One partner stated, “This helped me gain experience in conducting CBPR.”

### Dissemination and Sustainability

Regarding data ownership and participation in dissemination activities, one partner put it, “The research was translational, and information was provided to the community. It helped me to work with the community in a different capacity, giving me a different perspective.” Another partner emphasized, “While I have had experience running a research team, it was with training wheels. This opportunity allowed me to write a grant, run a research team, and manage writing the manuscripts for publication.” Yet another partner further delved into this point, stating, “We made collective decisions; the community came up with the questions, went out and collected the data.” Sustainability was an emerging challenge at the project’s end, as maintaining partnerships was crucial. Partnerships needed additional funding to continue their work in the communities, which would also help them sustain and maintain their relationships. As highlighted by one partner, “Getting funding to continue is important to give back to the community.” To sustain relationships, we maintained a network of partners and facilitated communication within the network based on the needs identified. We used emails, newsletters, and other means of communication to share information and resources on securing external funding with the network and continued to offer technical assistance and other forms of support for securing funding. One partner noted, “This grant has expanded my research agenda and will make me more competitive for additional grants. The funding has also provided me with an opportunity to train more students.”

## Discussion

Seed funding is a critical element for initiating CBPR projects. Some studies have emphasized the value of seed funding alone (Main et al., 2012; Thompson et al., 2010), while others have suggested that it should be complemented by additional support (Coombe et al., 2023; Jenkins et al., 2020; Kegler et al., 2016). Our research findings underline the importance of providing comprehensive services for recipients of such awards, tailored to the specific stage of readiness of the partners. As supported by the literature, the evaluation of the CBPR Small Grants Program has revealed that relationship building, role negotiation, trust, power distribution, and decision-making are key elements of the partnership development process and the overall success of the project. (Coombe, C. M., 2023). Academic programs can support research partners, and research offices should update their processes to meet the needs of CBPR studies. Researchers from Virginia Commonwealth University propose using natural language processing and deep-learning algorithms to categorize Institutional Review Board protocols into five partnership categories: Non-Community Engaged Research, Instrumental, Academic-led, Cooperative, and Reciprocal (Zimmerman et al., 2022). This categorization can aid in identifying studies as Non-Community-Engaged Research and at a higher level of engagement than investigator-recorded data. Such an approach could help universities and research institutions track progress and coordinate efforts to meet community needs.

Partnership development is a crucial component of the CBPR process. For individuals new to CBPR, the Connection phase represents a critical entry point that requires careful attention to the needs and understanding of potential partners. Previous research highlights the multiple capacity-development and support services needed for novice CBPR investigators (Teufel-Shone et al., 2019)(Andrews et al., 2013)(Collins et al., 2023). Our experience, consistent with the literature, emphasizes the need for conscious efforts to support organic relationship formation, networking, and idea exchange well before discussing specific CBPR collaborations (Cleveland, 2014). Therefore, networking opportunities were provided to a broad range of stakeholders from various disciplines, experiences, and skills to increase the likelihood of future collaborations, peer-support, and re-entry into the CARES cycle. Unfortunately, academic centers and funding agencies often underinvest in the Connection stage, providing more support to applicants who have already formed formalized partnerships around a project or proposal (Coombe et al., 2020)(Israel et al., 2006). The CARES model highlights the importance of partners building rapport and engaging in conversations and negotiations during the Partnership Development stage before innovation and collaborative actions begin. Activities associated with the Connection phase pave the way for individuals who do not have existing partner prospects upon entering the program, a consistent challenge with risks involved, mimicking real-world scenarios where relationships are built organically based on mutual interests and agreed-upon pre-conditions.

At the Innovation stage, partners draw upon their individual experiences, ambitions, and expertise to co-develop a project that is mutually beneficial, especially for the target community, and agreed upon (Brush et al., 2011)(Ortiz et al., 2020)(Samuel et al., 2018). To ensure equitable partnerships, a common practice is to incorporate role and responsibility negotiations into the planning process (Winckler et al., 2013). Additionally, providing seed funding through small awards can strengthen and maintain momentum, while also piloting a program and collecting preliminary data (Brush et al., 2011; Coombe et al., 2020; Winckler et al., 2013). It is essential to note that regardless of the project size or grant amount, the CARES model assumes that partnership teams have secured funding for the plan formulated during the Innovation stage before progressing to the Collaborative Action stage.

### Collaborative Action and Sustainability in CBPR Partnerships

After successful partnership development and innovation, partners can put their collaborative plan into action. To ensure success, it is important to prompt reflection into the partnership process, which can lead to identifying the knowledge, skills, and resources needed to successfully carry out the plan. Previous studies have emphasized the need to clarify roles and concerns to prevent issues from arising that could affect the success of the project if left unaddressed (Wallerstein & Duran, 2006; Winckler et al., 2013; Coombe et al., 2020; Brush et al., 2011). While it is important to involve community members in discussions around research and analysis, the purpose here is not to teach those skills so that community partners can assume those roles. Our findings suggest that it may be more cost-effective to focus on empowering community and academic members to fulfill their respective roles rather than having community members become researchers and researchers assume the roles of community partners. A good strategy is to train partners in validated tools and methods to involve diverse groups in addressing issues. One example is the SEED Method, which is a participatory approach that can be adapted to develop strategies for reducing health problems, such as opioid misuse and overdoses, and implemented by community stakeholders in collaboration with a participatory research team (Zimmerman et al., 2020). What is important is to generate capacity for each partner to understand, appreciate, and support the incorporation of their unique worldview, resources, and knowledge into their collaboration. In the visioning workshop facilitated by the Engaged for Equity team, our partners created visuals based on the River of Life, resulting in insightful conversations about where they stand, what they want to achieve collectively, and how the program could support them along the way. Reflection could be encouraged through brief self-evaluation of the partnership, feeding into a co-authored project report both during implementation and at the conclusion of the project.

The final stage of CBPR collaborations usually involves dissemination efforts and planning for the future. Our partners were productive and participated in conferences, wrote manuscripts and other grants, and served as the inaugural members of Morgan CARES. Participation in advanced professional writing and other targeted skill-development sessions that support the co-development of educational materials, publications, and presentations are key factors in continuing the relationship, sharing the credit, and aiming for greater impact. Facilitating planning sessions for new partners to schedule their dissemination and plan for future activities helps illuminate pathways for continued engagement beyond the project and further strengthens the partnership. Sustainability is an overarching goal of CBPR, which produces long-lasting, meaningful impacts on communities through collaboration and long-term sustainability of programs and initiatives. Positive partnership experiences and continued funding are two significant predictors of sustainability and maintenance (Wallerstein & Duran, 2006; Coombe et al., 2020; Brush et al., 2011). However, some partnerships may not survive, if the issues are not addressed early on. One major challenge that can jeopardize CBPR projects is negative partnership experiences, such as imbalanced decision-making power, as revealed by the qualitative findings. Another reason could be related to funding difficulties, as evidenced by the qualitative themes, participant feedback, and the small number of projects that applied and secured subsequent funding. However, many of these issues could be prevented. Given that securing subsequent funding following a small grant is considered a measure of success and has been incorporated into CBPR programming previously, we suggest that programs implementing this model offer or facilitate access to supplemental funding for promising initiatives. Programs using the CARES Model could also fund dissemination activities and encourage partnership teams to co-develop professional writings and other publications, which has been noted by other programs as an important aspect of continued capacity building and success and contributes to partnership maintenance.

### Capacity Development

Capacity building is a crucial aspect of CBPR, and it involves training and technical assistance. Although several frameworks guide the implementation of CBPR, capacity building remains a central tenet. Participants in the CBPR Small Grants Program emphasized that capacity-building activities enhance the co-learning experience, as evidenced by qualitative feedback provided during follow-up and the workshops and technical assistance sessions provided in response to requests. These sessions can be customized to meet the required skill and knowledge level and to enhance the capacity of the whole partnership, contributing to the development of more effective projects.

To ensure the long-term orientation of capacity development, it is best to embed it in sustainable infrastructure where partner involvement happens organically, rather than through academically controlled didactic trainings and mentoring. As individuals and partnership teams master the skills and competencies relevant to CBPR approaches to health equity, they can become mentors for other less-experienced individuals and teams. Previous initiatives such as the Community Research Scholars Initiative (CRSI) in Cleveland, Ohio, have attempted to equalize power by providing intensive research training and mentoring to members of the community and community-based organizations (Collins et al., 2023). This approach to capacity building not only facilitates shared understanding but also validates the community partner as credible, which is noted in the literature as a perpetual challenge for community partners in research relationships. Overall, it is essential to prioritize capacity building as a fundamental component of CBPR and to continually assess and revise strategies for its effective implementation.

### Limitations

It is important to acknowledge the program’s limitations to fully understand its impact. First, the program was limited by its small sample size and the lack of long-term follow-up data. The findings from the program were confined to the context of one Historically Black College and University, making it difficult to generalize the results to other minority-serving institutions. Second, the program was evaluated through a formative evaluation model, using mostly unstructured qualitative evidence, meeting notes, project reports, etc. This meant that the evaluation was still being developed, refined, and the data were triangulated, while the program was being administered and the CARES model developed. This made it challenging to gather comprehensive data that could inform the program’s design and implementation. Nevertheless, the program’s formative evaluation has also offered valuable insights into the program’s strengths and weaknesses, which have been used to improve the program and inform the development of the network. Despite these limitations, the program’s weaknesses have become a catalyst for creating a robust network that can provide resources and support to the community and students. The challenges that the program faced have led to a more profound understanding of the unique needs and challenges faced by Historically Black Colleges and Universities, as well as the development of more effective strategies to address them. These weaknesses ultimately became a springboard for creating a more impactful and effective model. Despite these limitations, the ASCEND Small CBPR Grants Program has demonstrated a remarkable potential for creating a network that can empower and support minority-serving institutions and their students.

### Recommendations

Effective CBPR requires a robust infrastructure to support the partnership between community and academia. Without such support, CBPR projects can become disjointed, expensive, and less effective. This, in turn, can discourage junior researchers from getting involved and maintain the siloed nature of this work that has perpetuated historical mistrust between the community and academia. To address these issues, we recommend the following:

- Creating and maintaining an infrastructure that can facilitate community-academic partnerships and programs in a variety of settings. Such infrastructure should include access to resources, support for project management, and a mechanism for evaluating the effectiveness of the partnership.
- Developing an accredited certificate program that can provide community and academic partners with the credentials and skills they need to participate effectively in CBPR projects. Such a program can help to build trust between community partners and academia and ensure that the partnership is grounded in shared values and principles.
- Establishing non-profit, community-owned academic centers affiliated with universities. Such a center can serve as a hub for community-academic partnerships and provide the necessary resources and support to ensure that CBPR projects are effective, sustainable, and responsive to the needs of the community.

By implementing these recommendations, we may be able create a more supportive environment for CBPR initiatives and promote the development of strong, effective, and sustainable community-academic partnerships. These partnerships can help to break down silos between the community and academia and promote mutual respect, trust, and collaboration.

## Conclusion

Our CBPR Small Grants Program led to the development of the CARES model, a novel approach to guide community-academic collaborative projects to address health disparities. The model is flexible and adaptable to the changing needs and challenges of community-academic partnerships. It combines existing CBPR initiatives and practices with funding from our program to support community-academic collaborations. Adoption of the CARES model could help address the dearth of studies examining partnership processes independent of project outcomes. We call for further funding and support for CBPR partnerships to implement similar models and promote equitable research that reflects the needs of communities and improves health outcomes for all.

## Figures and Tables

**FIGURE 1.**
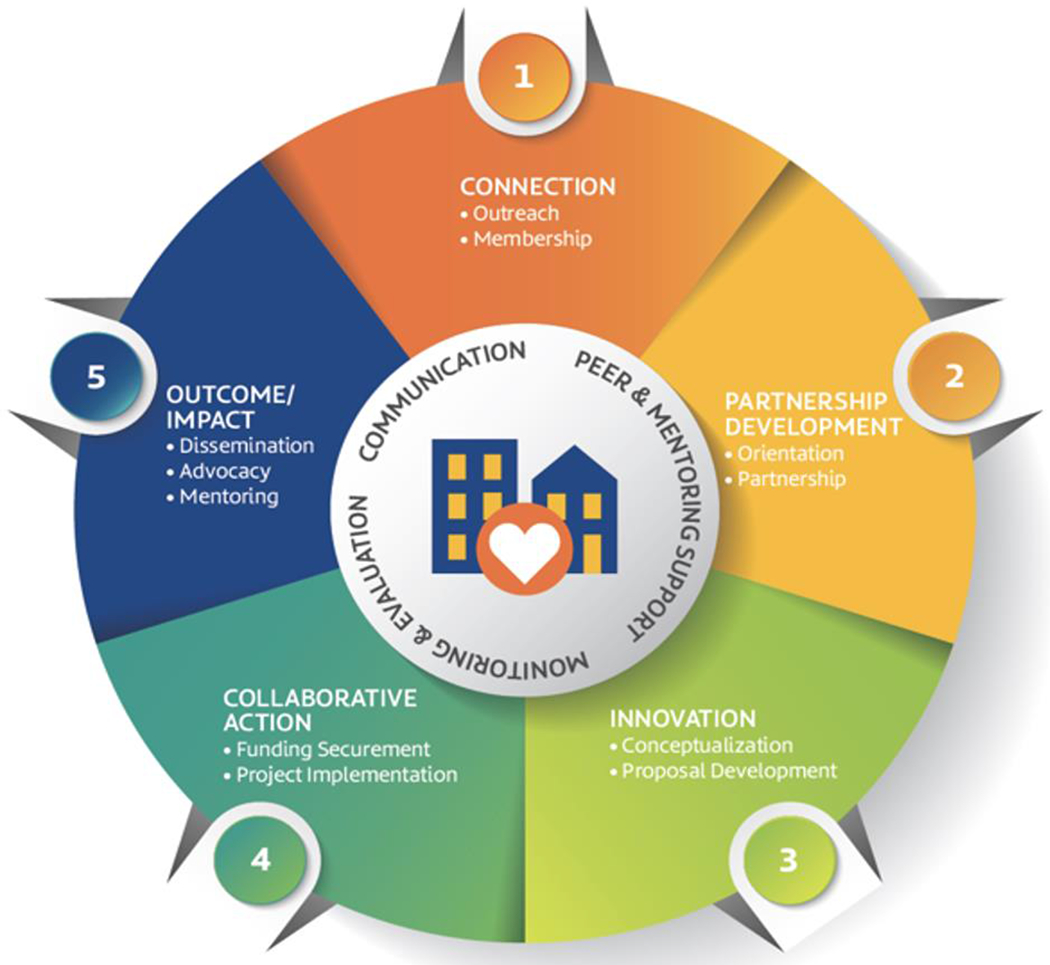
The CARES model.

**TABLE 1.** Characteristics of principal investigators (academic and community partners) of five of the fourteen funded projects

Characteristics	Academic (n=14)	Community (n=15)	Total (n=29)
Gender			
Female	11 (78.6%)	10 (66.7%)	21 (72.4%)
Male	3 (21.4%)	5 (33.3%)	8 (27.6%)
Race			
Black	8 (57.2%)	12 (80.0%)	20 (69.0%)
White	3 (21.4%)	2 (13.3%)	5 (17.2%)
Other	3^+^ (21.4%)	1^#^ (6.7%)	4 (13.8%)
Previous experience with CBPR	6 (42.9%)	4 (26.7%)	10 (34.5%)
Yes	8 (57.1%)	11 (73.3%)	19 (65.5%)
No			
Previous grant writing experience	10 (71.4%)	8 (53.3%)	18 (62.1%)
Yes	4 (28.6%)	7 (46.7%)	11 (37.9%)
No			

+ Asian: 2, Hawaiian: 1

# Hispanic/Latinx: 1

**TABLE 2.** Collaborative activities of funded project partnerships

Partnership Processes	# of principal investigators reporting
Shared mission and goals	Yes: 10 (71.4%)
	No: 4 (28.6%)
Partner involvement in project activities	Equal: 9 (64.3%)
	More academic: 4 (28.6%)
	More community: 1 (7.1%)
Decision making power	Equal: 6 (42.9%)
	More academic: 7 (50.0%)
	More community: 1 (7.1%)

**TABLE 3.** Identifying stages of the CBPR small grants program by mapping results of the qualitative evaluations and the main findings in terms of assets (A), perceived needs (N), and recommended responses (R) in each stage of readiness

Key objectives and Themes	Main Findings	Stage of readiness
Relationship building • Matchmaking • Roles and contributions	(A) Diverse perspectives and expertise of potential partners (N) Being introduced to and matched (finding and connecting) with partners that have complementary experiences and expertise (R) Networking opportunities and matchmaking services	Orientation and Connection
Technical assistance • Grant writing;	(A) Motivated to and interested in researching and solving the problems together (N) Guiding the process of co-developing the letters of intent, proposals, project budgets, etc. (R) Proposal-writing workshops and technical assistance	Ideation and Innovation
Partnership development • Building trust • Mutual benefits	(A) Diverse perspectives of partners and their complementary knowledge and competencies (N) Synergizing differing motives and creating a shared vision (N) Balancing power and control (R) Plenary discussions, reflection, and technical assistance	Collaboration
Co-ownership of results • Publications • Presentations	(A) Data collected and projects completed (N) Dissemination of the results among different audiences (R) Advanced training on professional writing, communications, and dissemination	Results and Dissemination
Sustainability • Mentoring • External funding	(A) More experienced and knowledgeable partners (N) Looking for funding to continue collaboration (R) Support partnership maintenance and growth by facilitating communication, sharing of resources, and co-learning (R) Advanced training and mentoring (R) Support for external funding (letters of support)	Maintenance and Growth

Capacity Development and Training
